# The association between the lymph node ratio, surgical margin, and survival in patients with colon cancer receiving adjuvant chemotherapy

**DOI:** 10.12669/pjms.38.3.5085

**Published:** 2022

**Authors:** Evren Fidan, Elif Merev, Arif Usta, Celal Alandag, Safak Disli, Ali Ozdover

**Affiliations:** 1 Dr. Evren Fidan, MD, Associate Professor Department of Medical Oncology, Faculty of Medicine, Karadeniz Technical University, Trabzon, Turkey; 2 Dr. Elif Merev, MD, Specialist, Department of Medical Oncology, Faculty of Medicine, Karadeniz Technical University, Trabzon, Turkey; 3 Dr. Arif Usta, MD, Assistant Professor, Department of General Surgery, Faculty of Medicine, Karadeniz Technical University, Trabzon, Turkey; 4 Dr. Celal Alandag. MD, Specialist, Department of Medical Oncology, Faculty of Medicine, Karadeniz Technical University, Trabzon, Turkey; 5 Dr. Safak Disli, MD, Specialist, Department of Medical Oncology, Faculty of Medicine, Karadeniz Technical University, Trabzon, Turkey; 6 Dr. Ali Ozdover, MD, Specialist, Department of Medical Oncology, Faculty of Medicine, Karadeniz Technical University, Trabzon, Turkey

**Keywords:** lymph nodes, colon cancer, surgical margins

## Abstract

**Objectives:**

To investigate the prognostic significant of lymph node ratio and surgical margins in patients with colon cancer undergoing surgery.

**Methods:**

This observational and retrospective study was conducted at Karadeniz Technical University Medical Faculty, between 1 January, 2010 and 31 November, 2020. A series of 137 patients who had undergone surgical resection of colon carcinoma were included in this study. mLNR, defined as the ratio of the number of mLNs to the number of examined lymph nodes, was calculated in colorectal cancer cases with lymph node metastasis. Patients were divided into three groups; LNR1 (< 0.25), LNR2 (0.25-0.6) and LNR3 (> 0.6).

**Results:**

Mean disease-free survival was 79.2 months (95% CI 71.0-87.4). The mean expected survival time was 73.5 months (95% CI: 65.9-81.0). As the metastatic LN ratio increased, the rate of local recurrence or distant metastasis increased statistically significantly (p=0.007). As the metastatic LN ratio increased, the death rate increased statistically significantly (p=0.036). Metastatic lymph node ratio did not have a statistically significant effect on overall survival in patients with stage-3 and more than 12 LNs removed (p=0.069). There was no statistically significant association between the closeness of the surgical margin and disease-free survival in stage 1 (p=0.505) and stage-2 (p=0.161). There was no statistically significant association between the closeness of the surgical margin and overall survival among patients with stage 1 (p=0.494) and stage 2 (p=0.265).

**Conclusion:**

A high metastatic LNR is associated with poorer overall and disease-free survival.

## INTRODUCTION

Colon cancer is one of the most common cancer in women and men, and represents approximately 1/3 of gastrointestinal system cancers.^1^ Lymph node involvement is associated with the prognosis of the disease and is one of the risk factors employed in the planning of adjuvant therapy^2^. Regional lymph nodes (pN) are classified solely on the basis of the number of lymph nodes involved. If lymph node involvement is present and there is no distant metastasis, patients are classified as stage III. Current guidelines state that 12 lymph nodes are sufficient for the assessment of lymph node involvement.^3^,^4^ However, insufficient lymph node removal can lead to pN-related problems and to difficulties in determining prognosis and treatment planning.^5^

Studies therefore began investigating the metastatic lymph node ratio (mLNR). Berger et al. first published a paper regarding the prognostic importance of the lymph node ratio (LNR) in 2005.^6^,^7^ One study involving 305 patients determined that the LNR and presence of perineural invasion affected both overall and disease-free survival.^7^ Another study showed that LNR calculation was more advantageous in determining prognosis than classic pTNM staging.^8^

This study was designed to evaluate the prognostic effect of the mLNR. Whether the surgical margin would have any effect on prognosis in addition to the mLNR was also investigated.

## METHODS

A series of 137 patients who had undergone surgical resection of colon carcinoma were included in this study. Patients with multiple primary cancer, and with missing follow-up information were excluded. All cancers were defined according to the TNM Classification of Malignant Tumors, eighth edition. Age at diagnosis, sex, date of initial diagnosis, tumor differentiation, location of distant metastasis and cancer-specific survival (CSS) were retrieved from databases. The study was performed at the Karadeniz Technical University Medical Faculty, between 1 January, 2010, and 31 November, 2020. The research was conducted out in line with the principles of the Declaration of Helsinki and was approved by the local ethical committee (No. 2019/38).

**Fig.1:**
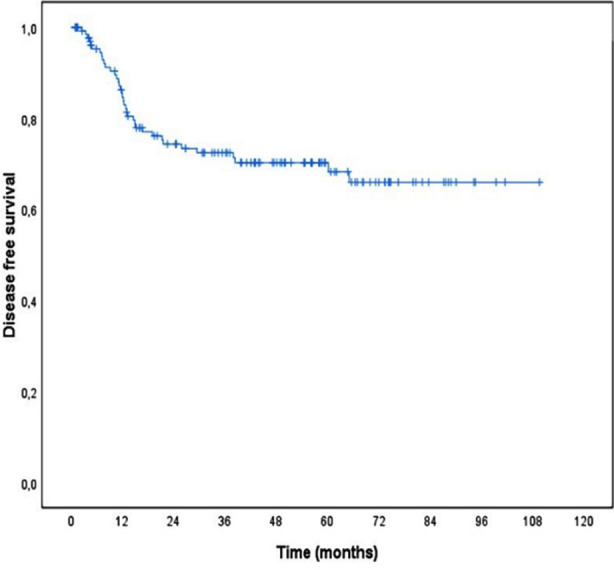
Disease-free survival of the patients.

### Definition and Evaluation of the Metastatic Lymph Node Ratio

mLNR, defined as the ratio of the number of mLNs to the number of examined lymph nodes, was calculated in colorectal cancer cases with lymph node metastasis. Patients were divided into three groups; LNR1 (< 0.25), LNR2 (0.25-0.6) and LNR3 (> 0.6).

### Statistical Analysis

Descriptive statistics for categorical data were expressed as numbers (n) and percentage (%) while quantitative data were given as mean ± SD and median (min-max). Kolmogorov-Smirnov test was used to investigate whether the normal distribution assumption was met. Whether the difference in CEA levels between pre- and post-op was statistically significant or not was evaluated Wilcoxon Sign Rank test. Kaplan-Meier survival curves were obtained both disease-free, and overall survivals. Cumulative survival rates for one, three and five years, mean expected duration of life and 95% confidence intervals were computed. Whether the potential factors were statistically significant effect on prognosis (i.e., disease-free, and overall survival) or not was investigated univariate Cox’s proportional hazard regression models. Multiple Cox’s proportional hazard regression models were obtained to determine the best independent predictors which mostly affected on prognosis. Any variable whose univariable test had a p value <0.25 was accepted as a candidate for the multivariable model. *Hazard ratios (HR) and*, 95% confidence intervals (CI) for each independent variable were also calculated. Data analysis was performed using IBM SPSS Statistics version 25.0 software (IBM Corporation, Armonk, NY, USA). A p value less than 0.05 was considered statistically significant.

**Fig.2:**
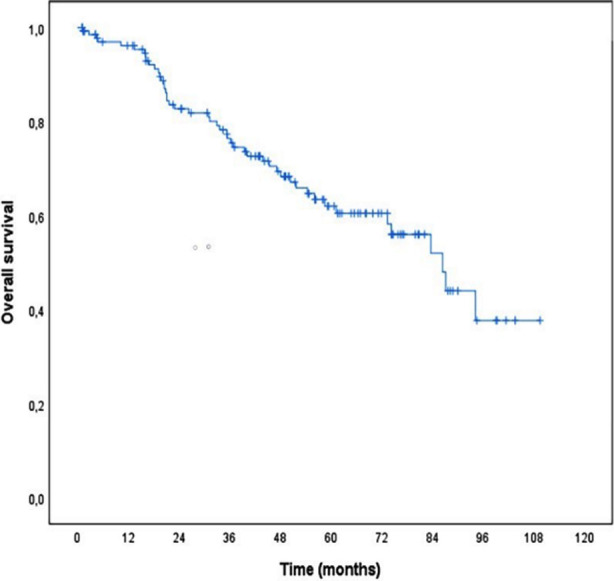
Overall survival of the patients.

## RESULTS

One hundred thirty-seven patients, 73 men and 64 women, and a mean age of 62.8±13.2 were included in the study. Patients’ clinicopathological parameters are summarized in Table-I and Table 2. Seventeen patients were stage-1, 64 were stage-2, and 56 were stage-3. Fifty-two patients had received adjuvant therapy, and 77 had not received it (Table-III). Local recurrence was observed in 15 (10.9%) of these patients and distant metastasis in 22 (16.1%).

**Table I:** Demographic and clinical characteristics of the patients.

	n=137
Age (year)	62.8±13.2
Age range (years)	28-97
***Gender***	
Female	64 (46.7%)
Male	73 (53.3%)
***Pathology***	
Adenocarcinoma	134 (97.8%)
Signet ring cell carcinoma	3 (2.2%)
Tumor diameter (cm)	5.0 (0.5 – 13.0)
Tumor size ≥5 cm	68 (49.6%)
***Tumor location***	
Ascending colon	66 (48.2%)
Transverse colon	7 (5.1%)
Descending colon	38 (27.7%)
Sigmoid colon	26 (19.0%)
Local recurrence	15 (10.9%)
Distant metastasis	22 (16.1%)
***Status***	
Alive	89 (65.0%)
Exitus	48 (35.0%)
Follow-up time (months)	43.9 (1.1 – 109.6)

**Table II:** Other clinical features of the patients.

	Descriptive statistics
***T stage***	
T1	4 (3.0%)
T2	14 (10.4%)
T3	104 (77.0%)
T4	13 (9.6%)
***Grade***	
1	72 (74.2%)
2	17 (17.6%)
3	8 (8.2%)
***LVI***	
No	68 (76.4%)
Yes	21 (23.6%)
Proximity to surgical margin	50.0 (0.0 – 230.0)
Number of LAPs removed	14.0 (1.0 – 54.0)
Removed LAP >12	79 (57.7%)
Metastatic LAP	0.0 (0.0 – 11.0)
***Metastatik LN oranı***	
0.00-24.99%	120 (87.6%)
25.00-60.00%	11 (8.0%)
>60.00%	6 (4.4%)
***Stage***	
1	17 (12.4%)
2	64 (46.7%)
3	56 (40.9%)

**Table III:** Frequency distribution of the cases in terms of adjuvant therapy.

	Descriptive statistics
***Adjuvant treatment***	
Recieved	52 (40.3%)
Not recieved	77 (59.7%)
***Adjuvant treatment protocol***	
Capecitabine-oxaliplatin	20 (39.2%)
Capecitabine	8 (15.7%)
Fluorouracil-folinic acid	16 (31.4%)
Fluorouracil-folinic acid-oxaliplatin	7 (13.7%)
Number of cycles	6.0 (1.0 – 9.0)
***Number of cycles***	
≤3	10 (19.2%)
>3	42 (80.8%)

Mean disease-free survival was 79.2 months (95% CI 71.0-87.4). 1-year, 3-year and 5- year disease- free survival rates of the cases were 86.3%, 72.5% and 70.3%, respectively. Univariate and multivariate effects of all possible factors thought to be effective on disease- free survival were analysed by Cox’s proportional hazards methods (Table-IV). As a result of univariate statistical analyzes, the rate of local recurrence or distant metastasis increased statistically with the increase in the depth of tumor invasion, the presence of LVI, the presence of a small number of LNs or 12 or fewer LNs, an increase in the number of metastatic LNs and an increase in the rate of metastatic LN (p<0.05). In the next step, the factor(s) that had the greatest determinant on disease-free survival was determined by constructing the proportional hazard regression model of the multivariate Cox. The LVI variable with missing data problems was not included in the regression model. In addition, only the metastatic LN rate was included in the multivariate model, as there was a multicollinearity problem between the number of metastatic LNs and the rate of metastatic LN. According to the proportional hazard regression model of the multivariate Cox, the factors that most determine disease-free survival are respectively; metastatic LN ratio, number of LNs removed, and tumor invasion depth. As the rate of metastatic LN increased, the rate of local recurrence or distant metastasis increased statistically significantly (HR=1.023, 95% CI: 1.006-1.040, p=0.007). As the number of LNs removed increased, the rate of local recurrence or distant metastasis decreased statistically (HR=0.937, 95% CI: 0.893-0.983, p=0.008).

**Table IV:** Univariate and multivariate effects of all possible factors thought to be effective on disease-free survival.

	Univariate		Multivariate	
	
	HR (95% CI)	p-value	HR (95% CI)	p-value
Age	1.012 (0.988-1.037)	0.323	-	-
Male gender	0.883 (0.463-1.685)	0.706	-	-
Tumor diameter	0.948 (0.818-1.099)	0.480	-	-
Tumor size ≥5 cm	0.992 (0.516-1.907)	0.980	-	-
Left colon tumor	1.140 (0.598-2.173)	0.691	-	-
T stage	2.061 (1.026-4.139)	0.042	3.205 (1.266-8.116)	0.014
Grade	0.893 (0.471-1.692)	0.727	-	-
LVI	2.525 (1.208-5.280)	0.014	-	-
Proximity to surgical margin	1.000 (0.993-1.008)	0.960	-	-
Number of LAPs removed	0.932 (0.889-0.977)	0.003	0.937 (0.893-0.983)	0.008
Number of LAPs removed ≤12	2.006 (1.046-3.850)	0.036	-	-
Metastatic LAP	1.144 (1.008-1.297)	0.037	-	-
Metastatic lymph node ratio	1.022 (1.010-1.036)	<0.001	1.023 (1.006-1.040)	0.007
Stage	1.390 (0.832-2.322)	0.209	0.473 (0.188-1.190)	0.112
Receiving adjuvant treatment	1.496 (0.783-2.858)	0.223	1.322 (0.646-2.705)	0.445

HR: Hazard ratio, CI: Confidence interval.

One, three and 5-year disease-free survival rates of the cases were 96.2%, 76.4%, 62.0%, respectively. The mean expected survival time was 73.5 months (95% CI: 65.9-81.0). Univariate and multivariate effects of all possible factors thought to affect overall survival were analyzed using Cox’s proportional hazards method (Table-V).

**Table V:** Univariate and multivariate effects of all possible factors thought to be effective on overall survival.

	Univariate		Multivariate	
	
	HR (95% CI)	p-value	HR (95% CI)	p-value
Age	1.041 (1.019-1.064)	<0.001	1.036 (1.012-1.061)	0.003
Male gender	1.213 (0.684-2.150)	0.508	-	-
Tumor diameter	1.014 (0.900-1.143)	0.817	-	-
Tumor size ≥5 cm	0.929 (0.523-1.649)	0.801	-	-
Left colon tumor	1.280 (0.723-2.269)	0.397	-	-
T stage	2.008 (1.097-3.676)	0.024	2.415 (1.086-5.371)	0.031
Grade	0.463 (0.213-1.005)	0.052	-	-
LVI	2.273 (1.120-4.611)	0.023	-	-
Proximity to surgical margin	0.999 (0.993-1.006)	0.823	-	-
Number of LAPs removed	0.911 (0.870-0.954)	<0.001	0.931 (0.888-0.975)	0.003
Number of LAPs removed ≤12	2.909 (1.608-5.264)	<0.001	-	-
Metastatic LAP	1.212 (1.076-1.365)	0.002	-	-
Metastatic lymph node ratio	1.021 (1.010-1.033)	<0.001	1.015 (1.001-1.029)	0.036
Stage	1.698 (1.059-2.722)	0.028	0.735 (0.360-1.503)	0.399
Receiving adjuvant treatment	1.155 (0.635-2.100)	0.637	-	-

Mortality rate increased statistically significantly with advanced age, increased depth of tumor invasion, presence of LVI, few LN removals or 12 or fewer LN removals, increased number of metastatic LNs, increased rate of metastatic LN, and progression of the stage (p<0.05). In the next step, the factor(s) that had the greatest effect on overall survival was determined by constructing a multivariate Cox proportional hazard regression model. According to the proportional hazard regression model of the multivariate Cox, the most determinant factors on overall survival are respectively; the number of LNs removed, age, depth of tumor invasion, and rate of metastatic LN. When adjusted for other factors, the death rate decreased statistically significant as the number of LNs removed increased (HR=0.931, 95% CI: 0.888-0.975, p=0.003). The death rate increased with advancing age, independent of other factors (HR=1.036, 95% CI: 1.012-1.061, p=0.003). Independent of other factors, each 1-stage increase in the degree of tumor invasion increased the death rate statistically by 2.415 (95% CI: 1.086-5.371) times (p=0.031). Finally, as the rate of metastatic LN increased, the death rate increased statistically significantly (HR=1.015, 95% CI: 1.001-1.029, p=0.036).

As a result of the evaluation made on 32 cases with complete preoperative and postoperative CEA measurements, a statistically significant decrease was observed in the postoperative CEA level compared to preoperatively (p<0.001). As the CEA level decreased after surgery compared to preoperatively, the rate of local recurrence or distant metastasis decreased statistically significantly (HR=0.942, 95% CI: 0.900-0.985, p=0.009). As the CEA level decreased after surgery compared to preoperatively, the mortality rate decreased statistically significantly (HR=0.967, 95% CI: 0.940-0.995, p=0.019).

In patients with stage 3 and more than 12 LNs removed, the rate of metastatic lymph node did not have a statistically significant effect on disease-free survival (HR=1,030, 95% CI: 0.991-1.070, p=0.131). There was no statistically significant association between the number of LNs removed and overall survival in patients with stage 3 (HR=0.949, 95% CI: 0.891-1.011, p=0.104). Metastatic lymph node ratio did not have a statistically significant effect on overall survival in patients with stage 3 and more than 12 LNs removed (HR=1.035, 95% CI: 0.997-1.074, p=0.069).

There was no statistically significant association between the closeness of the surgical margin and disease-free survival in stage 1 (HR=0.990, 95% CI: 0.962-1.019, p=0.505) and stage 2 (HR=1.008, 95% CI: 0.997-1.020, p=0.161). There was no statistically significant association between the closeness of the surgical margin and overall survival among patients with stage 1 (HR=0.977, 95% CI: 0.914-1.045, p=0.494) and stage 2 (HR=1.006, 95% CI: 0.996-1.016, p=0.265).

## DISCUSSION

Colorectal cancers are some of the most common forms of cancer, and surgery is the main treatment in most colon cancers.^4^ The number of lymph nodes removed during surgery and the number of metastatic lymph nodes affects prognosis and treatment indication.^9^ Patients’ lymph node numbers were evaluated in this study. A lymph node greater than 12 was associated with better overall and disease-free survival results. This suggests that the greater the number of lymph nodes removed, the better the prognosis will be, because the metastatic lymph node detection rate will also increase with the number of lymph nodes.^4^ Studies have been designed based on the idea that the LNR may represent an alternative to pathological lymph node numbers in determining prognosis. The LNR is calculated as the proportion of pathological lymph nodes to the total number of lymph nodes removed.^10^ Ali et al. reported that the LNR is a more effective prognostic factor than lymph node staging in patients with stage III colon cancer.^11^ However, Mohan et al. reported that the LNR was of no additional value to survival.^12^ Various cut-off values have been reported for LNR in the literature.^13^,^4^ The current study employed the median LNR value as a cut-off, and the results were examined in three groups. In our study, as the rate of metastatic lymph nodes increased, the rate of local recurrence and distant metastasis increased. In addition, it was found that the mortality rate increased statistically as the metastatic lymph rate increased. When subgroup analyzes were performed, metastatic lymph node rate was evaluated in patients with stage 3 and more than 12 lymph nodes removed. In this patient group, the effect of metastatic lymph node ratio on disease-free and overall survival could not be determined. These results, result may be related to the number of patients with stage 3 and more than 12 lymph nodes dissected.

Studies have shown that right-side colon tumors have poorer prognosis and responses to treatment that left-side tumors. Although the reason is not fully understood, this may be due to mutations being seen more frequently in right-side colon tumors.^14^,^15^ No difference in survival was determined between patients with right-and left-side tumors in the present study.

Lymphovascular invasion (LVI) is regarded as a prognostic indicator.^16^,^17^ Stage of the disease, bowel obstruction, and lymphovascular invasion in addition to local tumor invasion are risk factors in terms of local recurrence.^18^ In our study, it was determined that the presence of lymphovascular invasion had a negative effect on both disease-free and overall survival. High risk factors in the literature include T4 tumor, perforation, LVI, perineural invasion, dissection of less than 12 lymph nodes, high grade, positive margins, and obstruction. However, one study reported no association between surgical margin positivity, PNI or LVI and mortality.^19^ Lin et al. determined a distant metastasis rate of 61.5% in patients with surgical margins of 1 mm or less.^20^ Another study determined an inverse relationship between survival and a positive surgical margin in stage IIb/C and IIIA patients.^21^ In our study, the association of surgical margin proximity with disease-free and overall survival in cases with stages one and two was examined, but no statistically significant association was found.

In colon cancer, adjuvant therapy is started at the sixth or eighth week. Studies have also shown that starting chemotherapy after the eighth week results in adverse effects on overall and disease-free survival.^22^,^23^ However, we found that adjuvant treatment had no effect on survival and we thought that this effect might be related to the number of patients.

CEA is a cell adhesion molecule and its level is increased in patients with colorectal cancer. There are some studies on the postoperative CEA level in patients with operated colon cancer.^24^,^25^ We examined pre-op and post-op CEA levels in this study. As the CEA level decreased after surgery compared to preoperatively, the rate of local recurrence, distant metastasis, and the mortality rate decreased statistically significantly and in clinical practice, these findings may be usefull for predicting the survey.

### Limitation of this study

It includes its retrospective and non-randomized nature.

## CONCLUSION

A high metastatic LNR is associated with poorer overall and disease-free survival. Metastatic LNR can be used in determining prognosis.

### Author’s Contribution:

**EF:** Designing the paper, Writing.

**EM:** Designing the paper, Data collection.

**AU:** Data collection, Writing.

**CA:** Data collection, Editing.

**SD:** Data collection, Writing.

**AO:** Data collection, Editing.
